# A Decentralized Low-Chattering Sliding Mode Formation Flight Controller for a Swarm of UAVs

**DOI:** 10.3390/s20113094

**Published:** 2020-05-30

**Authors:** Thiago F. K. Cordeiro, João Y. Ishihara, Henrique C. Ferreira

**Affiliations:** 1 Gama Campus, University of Brasília, Brasília 72444-240, Brazil; thiagocordeiro@unb.br; 2 Department of Electrical Engineering, Darcy Ribeiro Campus, University of Brasília, Brasília 70910-900, Brazil; ishihara@ene.unb.br

**Keywords:** unmanned aerial vehicle, synchronized multi-agent formation, decentralized sliding mode control

## Abstract

In this paper, a nonlinear robust formation flight controller for a swarm of unmanned aerial vehicles (UAVs) is presented. It is based on the virtual leader approach and is capable of achieving and maintaining a formation with time-varying shape. By using a decentralized architecture, the local controller in each UAV uses information only from the UAV itself, its neighbors, and from the virtual leader. Also, a synchronization control objective provides a mechanism to weight between the fleet achieving the desired formation shape, that is, achieving the desired relative position between the UAVs, and each UAV achieving its desired absolute position. The use of a combination of a sliding mode controller and a low pass filter reduces the usual chattering effect, providing a smooth control signal while maintaining robustness. Simulation results show the effectiveness of the proposed decentralized controller.

## 1. Introduction

The use of an unmanned aerial vehicle (UAV) swarm brings several advantages in search and rescue, disaster monitoring, aerial mapping, traffic monitoring, reconnaissance missions, and surveillance [1,2,3]. A swarm of UAVs provides system redundancy, reconfiguration ability, and structure flexibility, being more effective, flexible, robust, and reliable than single vehicles [4,5]. The formation control is a critical task of attempting cooperation among UAVs. In general, a formation control problem is to find a coordination scheme to enable UAVs to reach and maintain some desired, possibly time-varying formation or group configuration [6].

In the view of communication networks, the existing formation control approaches can be classified into the centralized method, where a single controller is used to control the whole team based on the information from the whole team [7] and the decentralized method, where each team member generates its own control based on local information from its neighbors [1,2,4,8,9,10,11]. Centralized formation control can be a good strategy for a small team of UAVs. When considering a team with a large number of UAVs, the need for greater computational capacity and a large communication bandwidth would mandate a decentralized formation control [4].

The main structures considered for formation control of UAVs swarm are leader-follower, behavioral, and virtual structure/virtual leader [12,13]. In the leader-follower approach [3,6,10,14], a common leader is chosen and the rest of the agents are assigned as followers. The group leader broadcasts its position information to the followers who then begin to follow the leader at an offset. In the behavioral approach [15,16], several desired behaviors are prescribed for agents in this approach. Such desired behaviors may include cohesion, collision avoidance, obstacle avoidance. In the virtual structure/virtual leader approach [4,9,11], the entire formation is treated as a single rigid body. The virtual structure can evolve as a whole in a given direction with some given orientation and can maintain a rigid geometric relationship among multiple vehicles. In the virtual leader approach, the leader is a known virtual entity and its information can be made available for each agent software.

There are several control techniques used in UAV formation control, based on distinct premises and aiming to achieve distinct objectives. A common approach is to use a nonlinear dynamic inversion (NLDI) which, via nonlinear functions, encapsulates the nonlinear system in a box with virtual inputs/outputs that behaves as a linear system. This linearized system act as a set of double integrators, that then is controlled by any linear or nonlinear technique, such as pole placement [14,17], $H_{\infty }$ control [9], differential game approach [10] or sliding mode control (SMC) [8,18]. There are two main approaches when using the NLDI fixed-wing UAV formation flight control, related to in which frame the whole formation is described. One is to choose a global frame, such as north-east-down [9,10], and the virtual inputs are accelerations in north/south, east/west and up/down directions. Other is to use a leader related frame [8,14,17,18], and the virtual inputs accelerates toward forward/backward, left/right and above/below the leader.

In References [14,17], classic controllers were designed after applying of NLDI procedure in the nonlinear dynamics of UAVs formation flight. In Reference [10], a differential game approach is used to achieve an optimal controller that weights between minimizing the terminal position and velocity error of each UAV and minimizing the control effort. Another option, as in Reference [2], is the model predictive control (MPC), which can be used to compute an optimal control output to achieve formation control while avoiding obstacle and dealing with actuator saturation. It is however computationally expensive, since it reevaluates, at each time instant, the optimal control output over a finite time horizon. In Reference [2], the computational cost is partially reduced by maintaining the previously computed control output and reevaluating only when certain trigger events indicates that the control output must be changed, which works well in steady maneuvers, such as straight level flight or in constant-rate turns. In all of these approaches [2,10,14,17], the project does not account for the effect of disturbances or model uncertainty. Robust [8,9,11,18,19] and adaptive [1] approaches are appropriate for tackling this problem, where robust approaches usually has fast response but has a high control effort and/or chattering, whereas adaptive approach has a slower convergence, but uses a smoother control signal. In this way, the robust approach is recommended if precision of the formation is more important than control effort. In Reference [9], the proposed $H_{\infty }$ linear controller is robust to noises, disturbances, and delays in communication between the UAVs. The SMC is an interesting technique since it, ideally, can completely compensates the effects of model uncertainties and bounded disturbances. As disadvantage, it provides a discontinuous, chattering control signal whose source is a signum (sign) function [11,19,20]. A possible solution is to change this signum function to a saturation (sat) function, as in our previous work for UAV formation flight [18], but this generates a trade-off between precision and chattering. Another solution is the use of a second-order SMC (SOSMC) [21,22], which uses the integral of the chattering signal as control input of a plant. A generalization of the second-order SMC is the low-pass filter (LPF) [23,24,25,26]. The integral, or the more general low-pass filter, is included as part of the plant model, which improves precision. For example, in Reference [23] an architecture with sliding mode control and low pass filter is proposed for synchronous position control for multiple robotic manipulator systems. However, its control law involves the computation of derivatives whose order is higher than the plant order, which can difficult implementation for example in an embedded system. In Reference [24] an attitude controller for the reentry of a space vehicle based on low pass filter SMC architecture is proposed. Differently from, for example, Reference [23], the LPF in Reference [24] is used to filter only the signal component that contains a signum function, while bypassing the smooth component of the control signal directly to the plant. This approach, compared to the approach in Reference [23], avoids the computation of higher-order derivatives.

In this paper, using an LPF-based SMC approach, a decentralized controller for a time-varying synchronized formation of multiple UAVs with a virtual leader is proposed. It is considered that each UAV is subject to unknown bounded disturbance. The computation of higher-order derivatives is not required. This is achieved by decomposing the control signal in smooth and in chattering components and filtering only the chattering component. Compared with Reference [24], which considers a single space vehicle, the proposed controller considers a synchronized and decentralized formation of multiple UAVs. In a synchronized formation, multiple UAVs simultaneously converge to desired positions. In comparison with Reference [23], which uses LPF SMC for synchronized position control for multiple robotic manipulator systems in a ring-link communication topology, the proposed controller not require computation of higher-order derivatives, a more general information exchange topology is adopted, and the problem of UAV formation flying is considered. Different from our previous work [18], the proposed controller uses an LPF for chattering attenuation. The finite-time convergence to a linear sliding surface is proven by introduction of an appropriated Lyapunov function candidate and simulation results show the effectiveness of the proposed control architecture.

To use the LPF-based SMC, the upper bound of the disturbance must be known. Most of this disturbance is better described in the wind frame of the aircraft. For example, a model uncertainty can affect the lift force computation. The difference between the true and computed lift forces is equivalent to a disturbance force applied in the lift direction. Similar discussion can be made of the thrust, drag and side forces. If the NLDI linearizes the system in the leader’s wind frame [8,14,17,18], this upper bound can be used directly, under the assumption that the leader’s wind frame is similar enough to the followers’ wind frames, as the fleet in formation flies approximated to the same direction. If, however, the NLDI linearizes the system in a global frame [9,10], the wind-frame-described upper bound must be translated to the global frame. The equations to translate the disturbance upper bound to the global frame are developed in this paper.

The main contribution is now summarized. A formation flight controller is developed that includes, in a single controller, the following characteristics:

- uses the robust sliding mode control technique [8,11,18,19];
- has low-chattering with low degradation in performance by the use of a low-pass filter modelled as a plant component [23,24,25,26];
- uses a variant of the LPF SMC that is mathematically and computationally simpler than the usual approach, and removes computation of higher-order derivatives [24];
- is a multi-agent decentralized/synchronous approach [18,23].

It is worth noting that each individual characteristic of the controller listed above has already been developed in other papers but, to the best knowledge of the authors, there is no controller that includes all characteristics in a single controller. It is also worth noting that, to include all characteristics in a single controller, an appropriate Lyapunov that unifies theses characteristics is developed.

As a second contribution, a set of equations that translate the disturbance upper bound and is derivative upper bound from the wind frame to the global frame is proposed. These equations are used in the proposed LPF-SMC, but can be used, with minor modifications, in most fixed-wing formation SMC or SOSMC that are described in a global frame.

The remainder of this paper is organized as follows. Section 2 defines the problem, presents the mathematical models for the UAVs, formation flight, and communication graph. Section 3 presents the proposed controller, equations to compute the disturbance’s upper bound, and proves the stability of the controller. Section 4 evaluates the proposed controller by simulation against an unfiltered SMC, where it is shown that the controller significantly reduces its chattering without significantly reducing its performance, and Section 5 concludes this paper.

## 2. Preliminaries

In this section, models for an individual UAV and for a fleet formation are presented.

### 2.1. UAV Model

**Figure 1:**
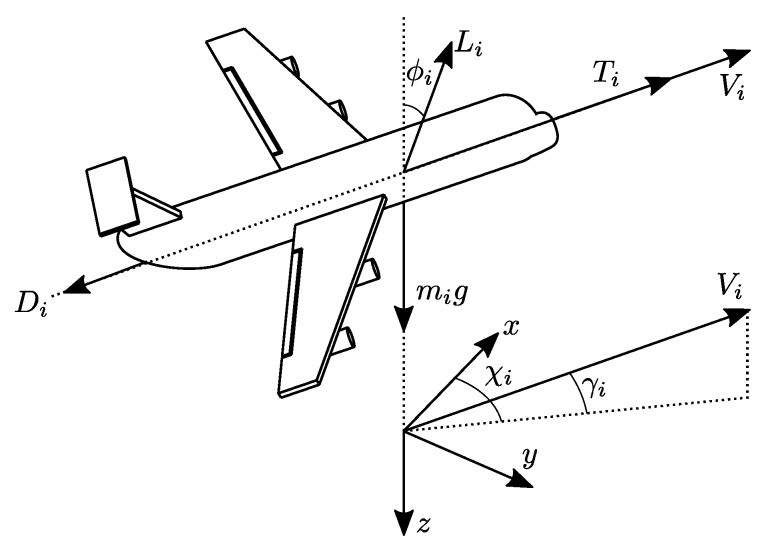
The *i*-th Unmanned Aerial Vehicle (UAV), its force and velocity vectors, and attitude angles.

The dynamics relating the input and output of the *i*-th vehicle in a fleet of *n* UAVs can be described by using the so-called point-mass aircraft model. It assumes a non-rotating flat Earth with a constant gravitational acceleration *g*. This model provides adequate precision to aircraft guidance and control problems and for short-range trajectory planning. It also assumes that the intensity of the wind is mild such that the airflow can be considered aligned with the vehicle fuselage, that is, that the angle-of-attack and sideslip angle are null, which are reasonable suppositions to cruise flight and coordinate maneuvers. Under these assumptions, as depicted in Figure 1, the drag force $D_{i}\left(t\right)$, generated by the airflow, is aligned to the fuselage, pointing backward, whereas the lift force $L_{i}\left(t\right)$ is perpendicular to the fuselage. It is assumed that the propulsive system provides a thrust force vector $T_{i}\left(t\right)$ aligned with the fuselage/airflow and in the opposite direction to $D_{i}\left(t\right)$ and that the net angular momentum generated by the propulsive system is null. Finally, it is assumed that the vehicle mass $m_{i}$ is (approximately) constant, i.e., the propulsive system is electric or if fuel-based, that the consumption is small compared to the total vehicle mass. These simplified suppositions are commonly used in the literature [9,10,27,28]. To achieve extra precision in more aggressive maneuvers, the effect of the angle-of-attack and sideslip angle should be included in the model [14]. In general, these quantities require a dedicated sensory system—which is usually not present in small UAVs—to these angles be measured. In this work, the angle-of-attack and sideslip angle are allowed to be unmeasured but it is supposed that their effect can be incorporated in the model as a bounded disturbance $b_{i}\left(t\right)$.

The state vector of the point-mass model of the *i*-th UAV is composed of its position vector $p_{i}\left(t\right)={\left[\;p_{xi}\left(t\right)\;p_{yi}\left(t\right)\;p_{zi}\left(t\right)\;\right]}^{T}$ described in the inertial Cartesian reference frame NED (North-East-Down) and by its velocity, described in a spherical coordinate system composed by the ground speed $V_{i}\left(t\right)$, flight path angle $\gamma_{i}\left(t\right)$ and course angle $\chi_{i}\left(t\right)$. By rotating the reference frame first by $\chi_{i}\left(t\right)$ around the *z* axis and after by $\gamma_{i}\left(t\right)$ around the rotated *y* axis, the *i*-th aircraft wind frame is obtained. Since it is assumed that the aircraft velocity vector is aligned with its fuselage, $\chi_{i}\left(t\right)$ and $\gamma_{i}\left(t\right)$ are equivalent respectively to the yaw $\psi_{i}\left(t\right)$ and pitch $\theta_{i}\left(t\right)$ attitude angles. The point-mass model includes also the roll attitude angle $\phi_{i}\left(t\right)$, which is a rotation of the fuselage around the direction of the velocity vector. The definition of the roll, pitch and yaw angles can be seen in [29]. Figure 1 shows the *i*-th UAV and its vectors and attitude angles.

The state change given by the derivative of $p_{i}\left(t\right)$ is computed as

(1) $${\dot{p}}_{i}\left(t\right)=\left[\begin{array}{c} {\dot{p}}_{xi}\left(t\right)\\ {\dot{p}}_{yi}\left(t\right)\\ {\dot{p}}_{zi}\left(t\right) \end{array}\right]=R_{i}\left(t\right)\left[\begin{array}{c} V_{i}\left(t\right)\\ 0\\ 0 \end{array}\right]=V_{i}\left(t\right)\left[\begin{array}{c} \cos\gamma_{i}\left(t\right)\cos\chi_{i}\left(t\right)\\ \cos\gamma_{i}\left(t\right)\sin\chi_{i}\left(t\right)\\ -\sin\gamma_{i}\left(t\right) \end{array}\right],$$

where

(2) $$R_{i}\left(t\right)=\left[\begin{array}{ccc} \cos\gamma_{i}\left(t\right)\cos\chi_{i}\left(t\right) & -\sin\chi_{i}\left(t\right) & \sin\gamma_{i}\left(t\right)\cos\chi_{i}\left(t\right)\\ \cos\gamma_{i}\left(t\right)\sin\chi_{i}\left(t\right) & \cos\chi_{i}\left(t\right) & \sin\gamma_{i}\left(t\right)\sin\chi_{i}\left(t\right)\\ -\sin\gamma_{i}\left(t\right) & 0 & \cos\gamma_{i}\left(t\right) \end{array}\right]$$

is a rotation matrix that rotates from the wind frame to the reference frame with angular velocity

(3) $$\omega_{i}\left(t\right)={\left[\;-{\dot{\chi }}_{i}\left(t\right)\sin\gamma_{i}\left(t\right)\;{\dot{\gamma }}_{i}\left(t\right)\;{\dot{\chi }}_{i}\left(t\right)\cos\gamma_{i}\left(t\right)\;\right]}^{T},$$

and the variables $V_{i}\left(t\right)$, $\chi_{i}\left(t\right)$, and $\gamma_{i}\left(t\right)$ can be computed by

(4) $$\tan\chi_{i}\left(t\right)=\frac{{\dot{p}}_{yi}\left(t\right)}{{\dot{p}}_{xi}\left(t\right)},\;\sin\gamma_{i}\left(t\right)=-\frac{{\dot{p}}_{zi}\left(t\right)}{V_{i}\left(t\right)},\;V_{i}^{2}\left(t\right)={\dot{p}}_{xi}^{2}\left(t\right)+{\dot{p}}_{yi}^{2}\left(t\right)+{\dot{p}}_{zi}^{2}\left(t\right).$$

The UAV dynamics is described by [9]

(5) $$\begin{array}{cc} {\dot{V}}_{i}\left(t\right) & =\frac{T_{i}\left(t\right)-D_{i}\left(t\right)}{m_{i}}-g\sin\gamma_{i}\left(t\right)+b_{ti}\left(t\right),\\ {\dot{\chi }}_{i}\left(t\right) & =\frac{L_{i}\left(t\right)\sin\phi_{i}\left(t\right)}{m_{i}V_{i}\left(t\right)\cos\gamma_{i}\left(t\right)}+\frac{b_{\psi i}\left(t\right)}{V_{i}\left(t\right)\cos\gamma_{i}\left(t\right)},\\ {\dot{\gamma }}_{i}\left(t\right) & =\frac{L_{i}\left(t\right)\cos\phi_{i}\left(t\right)}{m_{i}V_{i}\left(t\right)}-\frac{g\cos\gamma_{i}\left(t\right)}{V_{i}\left(t\right)}+\frac{b_{\theta i}\left(t\right)}{V_{i}\left(t\right)}, \end{array}$$

where the disturbance signal $b_{i}\left(t\right)=\left[\;b_{ti}\left(t\right)\;b_{\theta i}\left(t\right)\;b_{\psi i}\left(t\right)\;\right]$ encompasses model approximations, parameter uncertainty, and disturbances in acceleration, generated by several sources, such as wind. It is supposed that $b_{i}\left(t\right)$ and ${\dot{b}}_{i}\left(t\right)$ are unknown but with known bounds. The subscripts *t*, θ, and ψ from the elements of $b_{i}\left(t\right)$ means respectively thrust, pitch, and yaw. The thrust force magnitude $T_{i}$ is a function of the engine throttle; $D_{i}\left(t\right)$ is the magnitude of the drag force; the lift force magnitude $L_{i}$ is a function of several parameters, such as air density and aircraft speed, and is adjusted mainly by changing the elevator position and $\phi_{i}$ is adjusted by a combination of aileron and rudder positions. The variables $T_{i}\left(t\right)$, $L_{i}\left(t\right)$, and $\phi_{i}\left(t\right)$ are the control inputs at the *i*-th UAV. It can be seen that Equation (5) presents a singularity when $V_{i}\left(t\right)=0$. Fixed-wing vehicles must maintain non-null airspeed to maintain its lift. Assuming null or mild wind speed, the ground speed $V_{i}\left(t\right)$ is also non-null and this singularity does not occur. It can be seen that $\cos\gamma_{i}\left(t\right)=0$ also presents a singularity in Equation (5). This occurs only when the UAV is flying exactly in an up or down direction. However, this is not an achieved state, except in highly acrobatic vehicles.

By defining the load factor $n_{i}\left(t\right)$ as [28]

(6) $$n_{i}\left(t\right)≜\frac{L_{i}\left(t\right)}{m_{i}g},$$

and defining the following virtual control input

(7) $$\Gamma_{i}\left(t\right)=\left[\begin{array}{c} a_{ti}\left(t\right)\\ a_{\psi i}\left(t\right)\\ a_{\theta i}\left(t\right) \end{array}\right]=\left[\begin{array}{c} \frac{T_{i}\left(t\right)-D_{i}\left(t\right)}{m_{i}}\\ gn_{i}\left(t\right)\sin\phi_{i}\left(t\right)\\ gn_{i}\left(t\right)\cos\phi_{i}\left(t\right) \end{array}\right],$$

the dynamics Equation (5) can be rewritten as

(8) $$\begin{array}{cc} {\dot{V}}_{i}\left(t\right) & =a_{ti}\left(t\right)-g\sin\gamma_{i}\left(t\right)-b_{ti}\left(t\right),\\ {\dot{\chi }}_{i}\left(t\right) & =\frac{a_{\psi i}\left(t\right)-b_{\psi i}\left(t\right)}{V_{i}\left(t\right)\cos\gamma_{i}\left(t\right)},\\ {\dot{\gamma }}_{i}\left(t\right) & =\frac{a_{\theta i}\left(t\right)-g\cos\gamma_{i}\left(t\right)-b_{\theta i}\left(t\right)}{V_{i}\left(t\right)}. \end{array}$$

By deriving ${\dot{p}}_{i}\left(t\right)$ from Equation (1), and applying some manipulations, is obtained that

(9) $${\ddot{p}}_{i}\left(t\right)=R_{i}\left(t\right)\left(\Gamma_{i}\left(t\right)+b_{i}\left(t\right)\right)+g,$$

where $g={\left[\;0\;0\;g\;\right]}^{T}$ is the gravitational acceleration vector. By defining

(10) $$\begin{array}{cc} \tau_{i}\left(t\right) & ={\left[\;\tau_{xi}\left(t\right)\;\tau_{yi}\left(t\right)\;\tau_{zi}\left(t\right)\;\right]}^{T}≜R_{i}\left(t\right)\Gamma_{i}\left(t\right)+g, \end{array}$$

(11) $$\begin{array}{cc} d_{i}\left(t\right) & ={\left[\;d_{xi}\left(t\right)\;d_{yi}\left(t\right)\;d_{zi}\left(t\right)\;\right]}^{T},≜R_{i}\left(t\right)b_{i}\left(t\right), \end{array}$$

the dynamics are finally rewritten as

(12) $${\ddot{p}}_{i}\left(t\right)=\tau_{i}\left(t\right)+d_{i}\left(t\right),$$

where $\tau_{i}\left(t\right)\in R^{3}$ is a virtual controller input and $d_{i}\left(t\right)\in R^{3}$ is the virtual disturbance described in the reference frame.

For the controller design, the model given by Equation (12) will be used. Once the virtual control signals are known, the original variables can be obtained. Since for any rotation matrix, $R_{i}^{-1}\left(t\right)$ = $R_{i}^{T}\left(t\right)$, the virtual input $\Gamma_{i}\left(t\right)$ can always be obtained from $\tau_{i}\left(t\right)$ by

(13) $$\Gamma_{i}\left(t\right)=R_{i}^{T}\left(t\right)\left(\tau_{i}\left(t\right)-g\right),$$

and then $T_{i}\left(t\right)$, $n_{i}\left(t\right)$, $\phi_{i}\left(t\right)$ can be obtained from Equation (7), which can finally be used as the input of an inner loop controller that actuates over the engine and control surfaces [8].

### 2.2. UAV Formation

It is considered a formation of UAVs with a virtual leader scheme. The virtual leader is designated here as the 0-th UAV, and consists of a virtual point with a position $p_{0}\left(t\right)={\left[\;p_{x0}\left(t\right)\;p_{y0}\left(t\right)\;p_{z0}\left(t\right)\;\right]}^{T}$ in space, known by all UAVs, which describes a smooth trajectory as a function of time. The results of this work can also be used for a non-virtual leader configuration by assuming that the leader UAV can broadcast its position to all followers UAVs.

The fleet formation is planned by the generation of the desired position $p_{i}^{d}\left(t\right)={\left[\;p_{xi}^{d}\left(t\right)\;p_{yi}^{d}\left(t\right)\;p_{zi}^{d}\left(t\right)\;\right]}^{T}$ for the *i*-th UAV which is described as

(14) $$p_{i}^{d}\left(t\right)=p_{0}\left(t\right)+{\tilde{p}}_{i}\left(t\right),$$

where ${\tilde{p}}_{i}\left(t\right)={\left[\;{\tilde{p}}_{xi}\left(t\right)\;{\tilde{p}}_{yi}\left(t\right)\;{\tilde{p}}_{zi}\left(t\right)\;\right]}^{T}$ is the desired (time-varying) clearance, which is described in the reference frame.

To achieve a formation shape that rotates with the leader is interesting to describe the desired clearance ${\tilde{p}}_{i}\left(t\right)$ in the leader’s wind frame or any other frame related to the leader as

(15) $${\tilde{p}}_{i}\left(t\right)=R_{r}\left(t\right){\tilde{p}}_{i}^{r}\left(t\right),$$

where ${\tilde{p}}_{i}^{r}\left(t\right)$ is the clearance vector described in a leader’s frame, such as wind, and the formation rotation matrix $R_{r}\left(t\right)$ rotates from the leader’s frame to the reference frame. For example, by defining $R_{r}\left(t\right)=R_{0}\left(t\right)$ (see Equation (2) with i=0), it is achieved formation description aligned with the (virtual) leader’s trajectory as in, for example, Reference [8,9]. If, instead, $R_{r}\left(t\right)$ is defined as

(16) $$R_{r}\left(t\right)=R_{\chi }\left(t\right)≜\left[\begin{array}{ccc} \cos\chi_{0}\left(t\right) & -\sin\chi_{0}\left(t\right) & 0\\ \sin\chi_{0}\left(t\right) & \cos\chi_{0}\left(t\right) & 0\\ 0 & 0 & 1 \end{array}\right],$$

it is achieved a formation description aligned with the horizontal projection of the (virtual) leader’s trajectory, used in, for example, References [14,17].

Another option is to describe the formation using a leader’s frame defined by the attitude Euler angles yaw $\psi_{0}\left(t\right)$, pitch $\theta_{0}\left(t\right)$, and roll $\phi_{0}\left(t\right)$. This can be useful, for example, for maneuvers involving close interaction between the leader and the followers, such as to a boom-receptacle automatic aerial refueling. In this case, $R_{r}\left(t\right)=R_{b}\left(t\right)$, where [29]

(17) $$\begin{array}{cc} R_{b}\left(t\right)≜ & \\ & \left[\begin{array}{ccc} \cos\psi_{0}\cos\theta_{0} & \cos\psi_{0}\sin\theta_{0}\sin\phi_{0}-\sin\psi_{0}\cos\phi_{0} & \cos\psi_{0}\sin\theta_{0}\cos\phi_{0}+\sin\psi_{0}\sin\phi_{0}\\ \sin\psi_{0}\cos\theta_{0} & \sin\psi_{0}\sin\theta_{0}\sin\phi_{0}+\cos\psi_{0}\cos\phi_{0} & \sin\psi_{0}\sin\theta_{0}\cos\phi_{0}-\cos\psi_{0}\sin\phi_{0}\\ -\sin\theta_{0} & \cos\theta_{0}\sin\phi_{0} & \cos\theta_{0}\cos\phi_{0} \end{array}\right]. \end{array}$$

The derivatives of $p_{i}^{d}\left(t\right)$ in Equation (14) can be computed as

(18) $$\begin{array}{cc} {\dot{p}}_{i}^{d}\left(t\right) & ={\dot{p}}_{0}\left(t\right)+{\dot{\tilde{p}}}_{i}\left(t\right), \end{array}$$

(19) $$\begin{array}{cc} {\ddot{p}}_{i}^{d}\left(t\right) & ={\ddot{p}}_{0}\left(t\right)+{\ddot{\tilde{p}}}_{i}\left(t\right). \end{array}$$

Using the Theorem of Coriolis [29], the derivatives of ${\tilde{p}}_{i}\left(t\right)$ in Equation (15) can be computed as

(20) $$\begin{array}{cc} {\dot{\tilde{p}}}_{i}\left(t\right) & =R_{r}\left(t\right)\left[{\dot{\tilde{p}}}_{i}^{r}\left(t\right)+\omega_{r}\left(t\right)\times {\tilde{p}}_{i}^{r}\left(t\right)\right], \end{array}$$

(21) $$\begin{array}{cc} {\ddot{\tilde{p}}}_{i}\left(t\right) & =R_{r}\left(t\right)\left\{{\ddot{\tilde{p}}}_{i}^{r}\left(t\right)+2\omega_{r}\left(t\right)\times {\dot{\tilde{p}}}_{i}^{r}\left(t\right)+{\dot{\omega }}_{r}\left(t\right)\times {\tilde{p}}_{i}^{r}\left(t\right)+\omega_{r}\left(t\right)\times \left[\omega_{r}\left(t\right)\times {\tilde{p}}_{i}^{r}\left(t\right)\right]\right\}, \end{array}$$

where $\omega_{r}\left(t\right)$ is the angular velocity between the rotating leader’s frame and the reference frame and is given by

(22) $$\omega_{r}\left(t\right)=\left\{\begin{array}{cc} \mathrm{leader}’s\;\mathrm{gyro}\;\mathrm{measurements}, & \mathrm{if}\;R_{r}\left(t\right)=R_{b}\left(t\right),\\ {\left[\;-{\dot{\chi }}_{0}\sin\gamma_{0}\;{\dot{\gamma }}_{0}\;{\dot{\chi }}_{0}\cos\gamma_{0}\;\right]}^{T}, & \mathrm{if}\;R_{r}\left(t\right)=R_{0}\left(t\right),\\ {\left[\;0\;0\;{\dot{\chi }}_{0}\;\right]}^{T}, & \mathrm{if}\;R_{r}\left(t\right)=R_{\chi }\left(t\right). \end{array}\right.$$

It is worth noting that when using the non-virtual leader’s body frame, the angular velocity $\omega_{r}\left(t\right)$ is the body angular velocity, which can be directly measured by a gyro sensor at the leader. By using a non-virtual leader and any of the wind frame variants, the ground velocity obtained from a GPS sensor or from a navigation algorithm must be used. When using a virtual leader approach, its trajectory is smooth, pre-known, and artificially generated, in a way that $\omega_{r}\left(t\right)$ can be pre-computed analytically or numerically with arbitrary precision depending on how the trajectory is created.

### 2.3. Communication Graph

Each follower UAV can exchange data with their neighbors. The communication network is represented by an undirected graph, which means that, if an *i*-th UAV receives data from a *j*-th UAV, this means that the *j*-th UAV receives data from the *i*-th UAV. The set of the UAVs that are neighbors of the *i*-th UAV is defined as $N_{i}$.

The Laplacian matrix L represents the connectivity between the UAVs

(23) $$L_{ij}=\left\{\begin{array}{cc} -a_{ij}, & \mathrm{if}\;j\neq i\;\mathrm{and}\;j\in N_{i},\\ \sum_{k\in N_{i}}a_{ik}, & \mathrm{if}\;j=i,\\ 0, & \mathrm{otherwise}, \end{array}\right.$$

where $a_{ij}=0$ means that there is no communication between the *i*-th and *j*-th UAVs and $a_{ij}>0$ means that there is a communication link between the *i*-th and *j*-th UAVs, and the value of $a_{ij}$ is used as a weight to the control algorithm that is developed in this paper. If all UAVs are reachable, that is, if someone starts from any UAV and can achieve any-other UAV via the communication links, L is semidefinite positive.

In decentralized controllers, the weight that is given to the information present in the own *i*-th UAV is also described, which is given by the diagonal matrix Λ. The matrix H includes both the own weight and the neighborhood weight. These matrices are given by

(24) $$\begin{array}{cc} H & =\Lambda +L, \end{array}$$

(25) $$\begin{array}{cc} \Lambda & =\mathrm{diag}(\left[\;\lambda_{1}\;\ldots \;\lambda_{n}\;\right]). \end{array}$$

Note that since $\lambda_{1},\ldots ,\lambda_{n}>0$ and L is semidefinite positive, the matrix H is invertible.

### 2.4. Formation Tracking and Synchronization Errors

The tracking error of each aircraft $e_{i}\left(t\right)={\left[\;e_{xi}\left(t\right)\;e_{yi}\left(t\right)\;e_{zi}\left(t\right)\;\right]}^{T}\in R^{3}$, relative to a desired position in the reference frame, is defined as

(26) $$e_{i}\left(t\right)≜p_{i}\left(t\right)-p_{i}^{d}\left(t\right).$$

The synchronization error $\Delta e_{ij}\left(t\right)={\left[\;\Delta e_{xij}\left(t\right)\;\Delta e_{yij}\left(t\right)\;\Delta e_{zij}\left(t\right)\;\right]}^{T}\in R^{3}$, which can be seen as a relative position error between the UAVs, is defined as

(27) $$\Delta e_{ij}\left(t\right)≜e_{i}\left(t\right)-e_{j}\left(t\right)=p_{i}\left(t\right)-{\tilde{p}}_{i}\left(t\right)-\left(p_{j}\left(t\right)-{\tilde{p}}_{j}\left(t\right)\right).$$

It can be seen that $\Delta e_{ij}\left(t\right)$ can be computed without knowing the leader’s position. However, since the computation of ${\tilde{p}}_{i}\left(t\right)$ and ${\tilde{p}}_{j}\left(t\right)$ in Equation (15) can be chosen to be dependent on the leader’s flight direction or attitude angles, it is assumed here that the leader’s data is available to all UAVs.

It is assumed that each *i*-th UAV can communicate only with a correspondent set of neighbor UAVs, $N_{i}\subset \left\{1,2,\ldots ,n\right\}$. The communication graph is assumed to be undirected, connected, not change with time, and previously known. Each UAV receives the tracking error information of other UAVs in the fleet only through its neighbors (as, for example, in the simulation in Section 4). The virtual leader can be seen as an extra node in the graph, that connects to every other UAV in a directed way, from leader to each follower.

The coupled error at *i*-th UAV is defined as the weighted sum of its tracking error and the synchronization error with respect to its neighbors, that is,

(28) $$e_{i}^{c}\left(t\right)={\left[\;e_{xi}^{c}\left(t\right)\;e_{yi}^{c}\left(t\right)\;e_{zi}^{c}\left(t\right)\;\right]}^{T}≜\lambda_{i}e_{i}\left(t\right)+\sum_{j\in N_{i}}a_{ij}\Delta e_{ij}\left(t\right)=\lambda_{i}e_{i}\left(t\right)+\sum_{j=1}^{n}a_{ij}\Delta e_{ij}\left(t\right),$$

in which $\lambda_{i}>0$ weights its own tracking error and $a_{ij}>0$ weights the error difference between the neighbor UAV *j* of the UAV *i*. In the last equality in Equation (28), if $j\notin N_{i}$ then $a_{ij}=0$. The synchronization control objective is to make the coupled errors approach to zero.

### 2.5. A Componentwise Formation Description

It is supposed that each component of $d_{i}\left(t\right)$ is independent of each other which implies that each component of ${\ddot{p}}_{i}\left(t\right)$ is independent of each other. In this way, the controller design is simplified since the description of only one axis is sufficient. A controller policy can be developed to a single axis and then it can be directly applied to the other two.

The one-dimensional dynamics from axis l=x,y,z, of the reference frame, is obtained from Equation (12) as

(29) $${\ddot{p}}_{li}\left(t\right)=\tau_{li}\left(t\right)+d_{li}\left(t\right).$$

Accordingly, the coupled tracking-synchronization error is obtained from Equation (28) as

(30) $$e_{li}^{c}\left(t\right)=\lambda_{i}e_{li}\left(t\right)+\sum_{j\in N_{i}}a_{ij}\left[e_{li}\left(t\right)-e_{lj}\left(t\right)\right].$$

## 3. Proposed Controller

**Figure 2:**
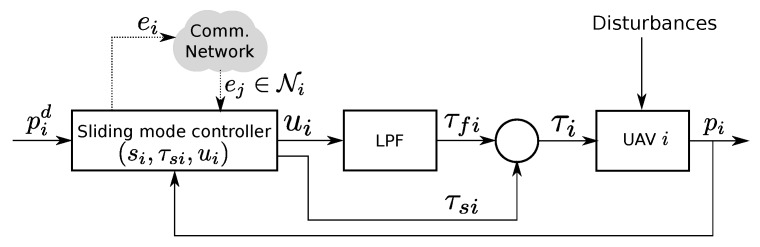
Block diagram of the control structure.

Here, a synchronous sliding mode controller is proposed. Figure 2 shows the proposed control structure. It achieves robustness against model uncertainty and disturbance. The chattering is attenuated by the use of a low pass filter (LPF).

To achieve synchronization, each UAV uses tracking errors of its neighbors to compute a sliding surface in the coupled error space. The sliding surface at the *i*-th UAV for the *l* axis is defined as

(31) $$s_{li}\left(t\right)={\ddot{e}}_{li}^{c}\left(t\right)+k_{d}{\dot{e}}_{li}^{c}\left(t\right)+k_{p}e_{li}^{c}\left(t\right).$$

As usual for sliding mode controllers, it is shown in the next subsection that $s_{li}\left(t\right)$ converges to zero in finite time, and maintains equal to zero thereafter. On the sliding surface, that is, when $s_{li}\left(t\right)=0$, the coupled error behaves according to the linear system

(32) $${\ddot{e}}_{li}^{c}\left(t\right)+k_{d}{\dot{e}}_{li}^{c}\left(t\right)+k_{p}e_{li}^{c}\left(t\right)=0,$$

which has all poles in the left plane and, thereafter, is exponentially asymptotically stable for project parameters $k_{d},k_{p}>0$.

The proposed control law for *i*-th UAV is

(33) $$\tau_{li}\left(t\right)=\tau_{li}^{s}\left(t\right)+\tau_{li}^{f}\left(t\right),$$

where $\tau_{li}^{s}\left(t\right)$ and $\tau_{li}^{f}\left(t\right)$ are, respectively, a smooth signal and a filtered signal of the control law, computed by

(34) $$\begin{array}{cc} \tau_{li}^{s}\left(t\right) & ={\ddot{p}}_{li}^{d}\left(t\right)-k_{d}{\dot{e}}_{li}\left(t\right)-k_{p}e_{li}\left(t\right), \end{array}$$

(35) $$\begin{array}{cc} {\dot{\tau }}_{li}^{f}\left(t\right)+\xi_{i}\tau_{li}^{f}\left(t\right) & =u_{li}\left(t\right), \end{array}$$

(36) $$\begin{array}{cc} u_{li}\left(t\right) & =-\mathrm{sign}(s_{li}\left(t\right))\eta . \end{array}$$

Equation (35) defines a low pass filter with cutoff frequency $\xi_{i}>0$ that converts a chattering signal $u_{li}\left(t\right)$ to a smooth signal $\tau_{li}^{f}\left(t\right)$. The parameter η must be chosen by the designer to guarantee the stability of the overall system.

The proposed control law given by Equations (33)–(36) contains only information from the virtual leader (or from a broadcasting non-virtual leader), from the own *i*-th UAV, and from its neighborhood $N_{i}$. The neighborhood information is contained in $s_{li}\left(t\right)$, defined in Equation (31), which is a function of $e_{li}^{c}\left(t\right)$ from Equation (30), which is a function of the own local error $e_{li}\left(t\right)$ and the neighborhood errors $e_{lj}\left(t\right)$, $j\in N_{i}$.

**Remark** **1.** *The variables $k_{p}$ and $k_{d}$ define the natural frequency and damping factor of the 2nd order local sliding surface $s_{li}$ of the i-th UAV from Equation (31). As can be seen in [20], these gains also define a control bandwidth, which must be sufficiently small to account for, for example, to actuator dynamics. Since it is chosen the same gain $k_{p}$ and the same gain $k_{d}$ to all UAVs, it means that they have sliding surfaces that share the same control bandwidth. This is reasonable if all UAVs have similar physical, actuator, and aerodynamic characteristics. However, if there are distinct UAVs, the constants must be chosen to respect the control bandwidth of the UAV with the slowest dynamics.*

### 3.1. Disturbance Model

Measurement or computation errors and the effect of non-modeled dynamics are incorporated in the dynamics model, given by Equation (12), as a disturbance signal described in the reference frame, $d_{i}={\left[\;d_{xi}\;d_{yi}\;d_{zi}\;\right]}^{T}$. It is supposed that the controller has no access to $d_{i}$ but there are known upper bounds $\Delta_{xi}$, $\Delta_{yi}$ and $\Delta_{zi}$ on the magnitude of the components of $d_{i}$ and upper bounds ${\tilde{\Delta }}_{xi}$, ${\tilde{\Delta }}_{yi}$, and ${\tilde{\Delta }}_{zi}$ on the derivatives of the components of $d_{i}$, that is,

(37) $$\begin{array}{c} |d_{li}\left(t\right)|\leq \Delta_{li},\left|{\dot{d}}_{li}\left(t\right)\right|\leq {\tilde{\Delta }}_{li},\;l=\left\{x,y,z\right\}. \end{array}$$

These upper bounds are used to define the value of η in Equation (36), as explained in Section 3.2. As a contribution of this paper is shown that the upper bounds on the components in the reference frame coordinates can be computed from the upper bounds $\delta_{ti}$, $\delta_{\theta i}$, and $\delta_{\psi i}$ on the components of the disturbance signal in the wind frame $b_{i}\left(t\right)$,

(38) $$|b_{ti}\left(t\right)|\leq \delta_{ti},\;|b_{\theta i}\left(t\right)|\leq \delta_{\theta i},\;\left|b_{\psi i}\left(t\right)\right|\leq \delta_{\psi i},$$

and from the upper bounds ${\tilde{\delta }}_{ti}$, ${\tilde{\delta }}_{\theta i}$ and ${\tilde{\delta }}_{\psi i}$ for the

(39) $$|{\dot{b}}_{ti}\left(t\right)|\leq {\tilde{\delta }}_{ti},\;|{\dot{b}}_{\theta i}\left(t\right)|\leq {\tilde{\delta }}_{\theta i},\;\left|{\dot{b}}_{\psi i}\left(t\right)\right|\leq {\tilde{\delta }}_{\psi i}.$$

The wind frame components of the disturbances are more naturally obtained, for example, in description of imprecision in the computation of drag or thrust forces. Assume that there is an upper bound $\Omega_{i}$ for the *i*-th UAV angular velocity $\omega_{i}$ and define the bounds vectors $\delta_{i}≜{\left[\;\delta_{ti}\;\delta_{\theta i}\;\delta_{\psi i}\;\right]}^{T}$ and ${\tilde{\delta }}_{i}≜{\left[\;{\tilde{\delta }}_{ti}\;{\tilde{\delta }}_{\theta i}\;{\tilde{\delta }}_{\psi i}\;\right]}^{T}$. From Equation (11), it can be seen that

(40) $$|d_{li}\left(t\right)|\leq ∥d_{i}\left(t\right)∥=∥R_{i}\left(t\right)b_{i}\left(t\right)∥=∥b_{i}\left(t\right)∥\leq ∥\delta_{i}∥.$$

The upper bounds of each component of $d_{i}$ are

(41) $$\Delta_{xi}=\Delta_{yi}=\Delta_{zi}=∥\delta_{i}∥.$$

Since Equation (11) involves two frames in which one rotates related to the other, its derivative is obtained by using the Theorem of Coriolis [29]

(42) $$\dot{d_{i}}\left(t\right)=R_{i}\left(t\right)\left({\dot{b}}_{i}\left(t\right)+\omega_{i}\left(t\right)\times b_{i}\left(t\right)\right),$$

where $\dot{d_{i}}\left(t\right)$ contains two components. The first, ${\dot{b}}_{i}\left(t\right)$, is the derivative of the disturbance $b_{i}\left(t\right)$, as seen by the wind frame. The second, $\omega_{i}\left(t\right)\times b_{i}\left(t\right)$, is generated by the rotation of the wind frame related to the inertial frame. See that a constant disturbance in the wind frame is a varying disturbance in the inertial frame, because of its rotation. Finally, $R_{i}\left(t\right)$ is used to represent the sum of these components in the inertial frame.

For the bounds $\delta_{i}$, ${\tilde{\delta }}_{i}$, and $\Omega_{i}$, it is obtained

(43) $$|{\dot{d}}_{li}\left(t\right)|\leq ∥\dot{d_{i}}\left(t\right)∥\leq ∥{\dot{b}}_{i}\left(t\right)∥+∥\omega_{i}\left(t\right)\times b_{i}\left(t\right)∥\leq ∥{\tilde{\delta }}_{i}∥+∥\Omega_{i}∥∥\delta_{i}∥.$$

In this way,

(44) $${\tilde{\Delta }}_{xi}={\tilde{\Delta }}_{yi}={\tilde{\Delta }}_{zi}=∥{\tilde{\delta }}_{i}∥+∥\Omega_{i}∥∥\delta_{i}∥.$$

Equations (40) and (44) provide the upper bounds to the proposed controller.

### 3.2. Stability Proof

To analyze the overall fleet behavior, all local variables must be concatenated in vectors. Concatenating the positions $p_{i}$, virtual control inputs $\tau_{i}\left(t\right)$, and disturbances $d_{i}\left(t\right)$ from all UAVs of the fleet results in respectively $P\left(t\right)={\left[\;p_{1}^{T}\left(t\right)\;\ldots \;p_{n}^{T}\left(t\right)\;\right]}^{T}$, $\tau \left(t\right)={\left[\;\tau_{1}^{T}\left(t\right)\;\ldots \;\tau_{n}^{T}\left(t\right)\;\right]}^{T}$, and $D\left(t\right)={\left[\;d_{1}^{T}\left(t\right)\;\ldots \;d_{n}^{T}\left(t\right)\;\right]}^{T}$, all $R^{3n}$ vectors. In this way, the dynamics of the fleet of UAVs is given by concatenating Equation (29) as

(45) $$\ddot{P}\left(t\right)=\tau +D\left(t\right).$$

Similarly, the error and coupled error in *x* axis are $R^{n}$ vectors given by $E\left(t\right)={\left[\;e_{1}^{T}\left(t\right)\;\ldots \;e_{n}^{T}\left(t\right)\;\right]}^{T}$ and $E^{c}\left(t\right)={\left[\;e_{1}^{cT}\left(t\right)\;\ldots \;e_{n}^{cT}\left(t\right)\;\right]}^{T}$ which are related by

(46) $$E^{c}\left(t\right)=\left(H⊗I_{3}\right)E\left(t\right),$$

where ⊗ denotes the Kronecker product and matrix H is given by Equation (24). The concatenation of the *n* UAVs sliding surfaces $S\left(t\right)={\left[\;s_{1}^{T}\left(t\right)\;\ldots \;s_{n}^{T}\left(t\right)\;\right]}^{T}$ is obtained as

(47) $$S\left(t\right)={\ddot{E}}^{c}\left(t\right)+k_{d}{\dot{E}}^{c}\left(t\right)+k_{p}E^{c}\left(t\right)=\left(H⊗I_{3}\right)\left(\ddot{E}\left(t\right)+k_{d}\dot{E}\left(t\right)+k_{p}E\left(t\right)\right).$$

The proposed sliding mode control law is written as

(48) $$\tau \left(t\right)=\tau^{s}\left(t\right)+\tau^{f}\left(t\right),$$

where $\tau^{s}\left(t\right)$ and $\tau^{f}\left(t\right)$ are computed by

(49) $$\begin{array}{cc} \tau^{s}\left(t\right) & ={\ddot{P}}^{d}\left(t\right)-k_{d}\dot{E}\left(t\right)-k_{p}E\left(t\right), \end{array}$$

(50) $$\begin{array}{cc} {\dot{\tau }}^{f}\left(t\right)+\Xi \tau^{f}\left(t\right) & =U(t), \end{array}$$

(51) $$\begin{array}{cc} U(t) & =-\mathrm{diag}\left\{\frac{\eta }{|s_{li}\left(t\right)|}\right\}S\left(t\right), \end{array}$$

with $U\left(t\right)={\left[\;u_{1}^{T}\left(t\right)\;\ldots \;u_{n}^{T}\left(t\right)\;\right]}^{T}$, $u_{i}\left(t\right)={\left[\;u_{xi}\left(t\right)\;u_{yi}\left(t\right)\;u_{zi}\left(t\right)\;\right]}^{T}$, and $\Xi ≜\mathrm{diag}\left(\left[\;\xi_{1}\;\ldots \;\xi_{n}\;\right]\right)⊗I_{3}\in R^{3n\times 3n}$.

To analyze the fleet stability, the following Lyapunov functional candidate is proposed

(52) $$V\left(t\right)=\frac{1}{2}S^{T}\left(t\right){\left(H⊗I_{3}\right)}^{-1}S\left(t\right).$$

Note that, since H and $H⊗I_{3}$ are a positive definite matrix, $H^{-1}$ and ${\left(H⊗I_{3}\right)}^{-1}$ are also a positive definite matrix, so V(t) is always positive for S(t)≠0.

By using Equations (45), (48) and (49), the sliding surface given by Equation (47) can be rewritten as

(53) $$\begin{array}{cc} S(t) & =\left(H⊗I_{3}\right)\left(\ddot{P}\left(t\right)-{\ddot{P}}^{d}\left(t\right)+k_{d}\dot{E}\left(t\right)+k_{p}E\left(t\right)\right)\\ \; & =\left(H⊗I_{3}\right)\left(\tau^{s}\left(t\right)+\tau^{f}\left(t\right)+D\left(t\right)-{\ddot{P}}^{d}\left(t\right)+k_{d}\dot{E}\left(t\right)+k_{p}E\left(t\right)\right)\\ \; & =\left(H⊗I_{3}\right)\left(D\left(t\right)+\tau^{f}\left(t\right)\right). \end{array}$$

Since ${\left(H⊗I_{3}\right)}^{-1}$ is constant, the derivative of Equation (52) is

(54) $$\dot{V}\left(t\right)=S^{T}\left(t\right){\left(H⊗I_{3}\right)}^{-1}\dot{S}\left(t\right).$$

By deriving Equation (53) and after using Equation (50), $\dot{V}\left(t\right)$ is rewritten to

(55) $$\begin{array}{cc} \dot{V}\left(t\right) & =S^{T}\left(t\right)\left(\dot{D}\left(t\right)+{\dot{\tau }}^{f}\left(t\right)\right)\\ \; & =S^{T}\left(t\right)\dot{D}\left(t\right)-S^{T}\left(t\right)\Xi \tau^{f}\left(t\right)+S^{T}\left(t\right)U\left(t\right)\\ \; & =\sum_{i=1}^{n}\left(s_{i}^{T}\left(t\right){\dot{d}}_{i}\left(t\right)-\xi_{i}s_{i}^{T}\left(t\right)\tau_{i}^{f}\left(t\right)+s_{i}^{T}\left(t\right)u_{i}\right)\\ \; & =\sum_{i=1}^{n}\left[\sum_{l=\{x,y,z\}}(s_{li}{\dot{d}}_{li}-\xi_{i}s_{li}\tau_{li}^{f}-|s_{li}\left|\eta \right)\right]\\ \; & \leq \sum_{i=1}^{n}\left[\sum_{l=\{x,y,z\}}|s_{li}\left|(\right|{\dot{d}}_{li}|+\xi_{i}|\tau_{li}^{f}\left|-\eta \right)\right]. \end{array}$$

The upper bounds of the disturbance and its derivative are given, respectively, by $\Delta_{li}\geq \left|d_{li}\left(t\right)\right|$ and ${\tilde{\Delta }}_{li}\geq \left|{\dot{d}}_{li}\left(t\right)\right|$, which are computed by, respectively, Equations (41) and (44). It is shown in [24] that $|\tau_{li}^{f}\left(t\right)|\leq |d_{li}\left(t\right)|\leq \Delta_{li}$. By using these upper bounds in Equation (55), it can be seen that

(56) $$\dot{V}\left(t\right)\leq \sum_{i=1}^{n}\left[\sum_{l=\{x,y,z\}}\left|s_{li}\right|\left({\tilde{\Delta }}_{li}+\xi_{i}\Delta_{li}-\eta \right)\right].$$

By choosing η satisfying

(57) $$\eta \geq {\tilde{\Delta }}_{li}+\xi_{i}\Delta_{li}+\epsilon ,\;\forall i\in \left\{1,\ldots ,n\right\},\;,\forall l\in \left\{x,y,z\right\},$$

for some arbitrarily chosen constant ϵ>0, it is obtained

(58) $$\dot{V}\left(t\right)\leq -\sum_{i=1}^{n}\sum_{l=\{x,y,z\}}|s_{li}\left(t\right)|\epsilon =-\epsilon \sum_{i=1}^{n}\sum_{l=\{x,y,z\}}\left|s_{li}\left(t\right)\right|=-\epsilon {∥S(t)∥}_{1},$$

where ${∥S(t)∥}_{1}$ is the 1-norm of S(t). Using the fact that the 1-norm is greater than the Euclidean norm of the same vector, then

(59) $$\dot{V}\left(t\right)\leq -\epsilon ∥S\left(t\right)∥,$$

which means that V(t) and, therefore, S(t) go to zero in finite time [20]. On the sliding surface, the system behaves as a stable linear system given by Equation (32) and the error converges asymptotically to zero.

**Remark** **2.** *Note that the sliding surface given by Equation (47), when rewritten in Equation (53), is a function only of the disturbance D(t) and the output of the filter $\tau^{f}\left(t\right)$. This has two main implications:*

*1.*  *Since it is shown here that S(t)→0, it follows that $\tau^{f}\left(t\right)\to -D\left(t\right)$. In this way, $\tau^{f}\left(t\right)$ estimates and compensates disturbances. Since the effect of airflow is not aligned to the fuselage is a disturbance, the presence of a disturbance compensation shows that the wind effect can be neglected in the initial model if this effect has known bounds.*
*2.*  *If the disturbance is null at t=0, S(0)=0 if $\tau^{f}\left(0\right)=0$ and the system already starts in sliding condition. Similarly, if the known disturbance upper bound is relatively small, the system starts near the sliding surface and converges fast to the sliding surface.*

## 4. Simulation

**Figure 3:**
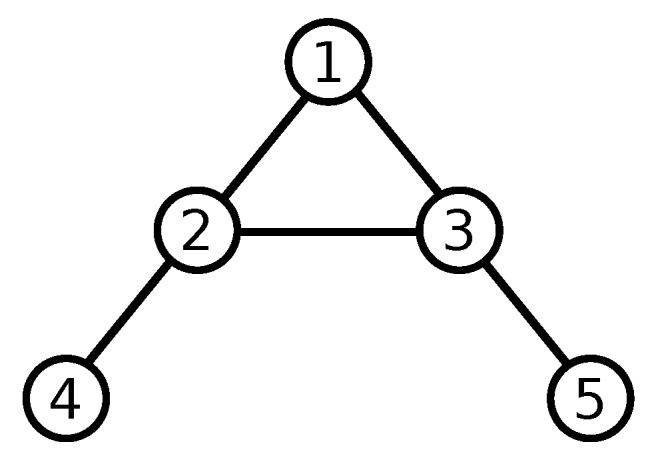
Five UAVs and their undirected communication links. The virtual leader is not shown here. All UAVs have access to the virtual leader’s trajectory information.

In this section, a simulation is made to show the effectiveness of the proposed controller. A scenario of 5 UAVs with communication links described by Figure 3 is used.

The matrices

(60) $$L=\left[\begin{array}{ccccc} 2 & -1 & -1 & 0 & 0\\ -1 & 3 & -1 & -1 & 0\\ -1 & -1 & 3 & 0 & -1\\ 0 & -1 & 0 & 1 & 0\\ 0 & 0 & -1 & 0 & 1 \end{array}\right]$$

and $\Lambda =I_{5}$ are chosen to give the same weight for the UAV own error and for each of its relative errors. The choice $k_{p}=0.5$ and $k_{d}=0.0625$ provide a critically damped sliding surface with natural frequency $\omega_{n}=0.25$ rad/s. These gains are chosen relatively small, as a way to limit the maximum commanded acceleration, even if the UAVs are initially far from their desired position. The low pass filters are settled such that $\Xi =I_{5}⊗I_{3}$.

A fleet with a non-rectilinear 3D trajectory is described, which is defined by the virtual leader path given by

(61) $$\left\{\begin{array}{c} p_{x0}\left(t\right)=80+45t\;\left[m\right],\\ p_{y0}\left(t\right)=20\cos\left(0.1t\right)\;\left[m\right],\\ \gamma_{0}\left(t\right)=\frac{\pi }{36}\;\mathrm{rad},\;\left(z_{0}\left(0\right)=-100\;m\right). \end{array}\right.$$

For easy visualization, a time-varying formation is considered, whose horizontal projection in the reference frame has a V-shape and the altitude has time-varying oscillation. Accordingly, the formation rotation matrix $R_{r}$ is defined as $R_{\chi }$ from Equation (16) and the clearance vectors ${\tilde{p}}_{i}^{r}\left(t\right)$ related to the virtual leader are

(62) $$\begin{array}{c} {\tilde{p}}_{1}^{r}\left(t\right)=\left[\begin{array}{c} 0\\ 0\\ 10\sin(0.1t) \end{array}\right],\;{\tilde{p}}_{2}^{r}\left(t\right)=\left[\begin{array}{c} -40\\ -40\\ 10\sin(0.1t+2\pi /5) \end{array}\right],\;{\tilde{p}}_{3}^{r}\left(t\right)=\left[\begin{array}{c} -40\\ 40\\ 10\sin(0.1t+4\pi /5) \end{array}\right],\\ {\tilde{p}}_{4}^{r}\left(t\right)=\left[\begin{array}{c} -80\\ -80\\ 10\sin(0.1t+6\pi /5) \end{array}\right],\;{\tilde{p}}_{5}^{r}\left(t\right)=\left[\begin{array}{c} -80\\ 80\\ 10\sin(0.1t+8\pi /5) \end{array}\right]. \end{array}$$

The initial position of each UAV is defined as

(63) $$\begin{array}{c} p_{1}\left(0\right)=\left[\begin{array}{c} 60\\ 0\\ -100 \end{array}\right],\;p_{2}\left(0\right)=\left[\begin{array}{c} 20\\ -30\\ -100 \end{array}\right],\;p_{3}\left(0\right)=\left[\begin{array}{c} 50\\ 20\\ -100 \end{array}\right],\;p_{4}\left(0\right)=\left[\begin{array}{c} 10\\ -50\\ -100 \end{array}\right],\;p_{5}\left(0\right)=\left[\begin{array}{c} 20\\ 80\\ -100 \end{array}\right]. \end{array}$$

The initial velocity of each UAV is defined as

(64) $$\begin{array}{c} {\dot{p}}_{1}\left(0\right)=\left[\begin{array}{c} 50\\ 5\\ 0 \end{array}\right],\;{\dot{p}}_{2}\left(0\right)=\left[\begin{array}{c} 40\\ 10\\ 0 \end{array}\right],\;{\dot{p}}_{3}\left(0\right)=\left[\begin{array}{c} 45\\ -10\\ 0 \end{array}\right],\;{\dot{p}}_{4}\left(0\right)=\left[\begin{array}{c} 40\\ -5\\ 0 \end{array}\right],\;{\dot{p}}_{5}\left(0\right)=\left[\begin{array}{c} 45\\ 0\\ 0 \end{array}\right]. \end{array}$$

The disturbance is simulated as

(65) $$b_{i}\left(t\right)=0.2{\left[\begin{array}{ccc} \cos(0.5t) & \cos(0.5t) & \cos(0.5t) \end{array}\right]}^{T},\;\forall i\in \left\{1,2,3,4,5\right\}.$$

From Equation (65), the magnitude of the upper bound vector $\delta_{i}$ of $b_{i}\left(t\right)$ is computed as $∥\delta_{i}∥=0.35$. The magnitude of the upper bound vector of ${\tilde{\delta }}_{i}$ is computed also from Equation (65) as $∥{\tilde{\delta }}_{i}∥=0.17$.

The upper bound of each component of $d_{i}\left(t\right)$ is computed by Equation (41) resulting in $\Delta_{xi}=\Delta_{yi}=\Delta_{zi}=0.35$. By simulation experiments it is verified that $\Omega_{i}=0.17$ rad/s is an upper bound for the angular velocity amplitude; the upper bound in ${\dot{d}}_{i}\left(t\right)$ is computed by Equation (44), resulting in ${\tilde{\Delta }}_{xi}={\tilde{\Delta }}_{yi}={\tilde{\Delta }}_{zi}=0.23$. By choosing ϵ=0.42, it is obtained from Equation (57) that η=1.

The system is implemented using an ode4 Runge-Kutta solver, with a fixed-step size of 1 ms. Since it is impossible to perfectly simulate the effect of a chattering input signal in a continuous differential equation, the controller output is evaluated at 10 ms time steps and maintained constant between time intervals.

For comparison purposes, the unfiltered synchronous formation flight controller presented in Reference [18] is also simulated. It is configured to be as similar as possible to the proposed controller. The first order sliding surface is defined with the same natural frequency as the proposed controller, that is, $\omega_{n}=0.25$ rad/s. By using the same upper bound $\Delta_{xi}=\Delta_{yi}=\Delta_{zi}=0.35$ and by choosing the same ϵ=0.42, it is computed η=0.77. Other parameters are exactly the same as the proposed controller.

**Figure 4:**
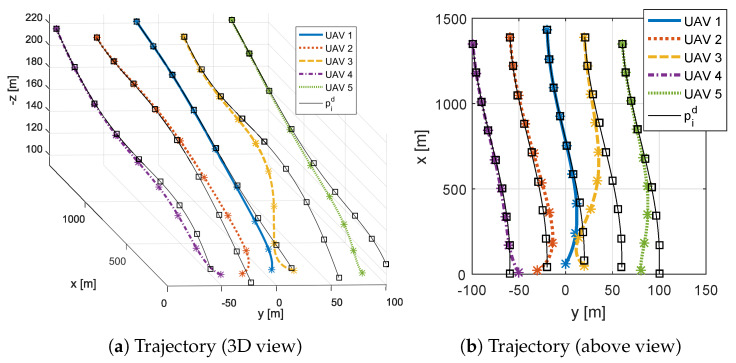
Desired trajectory and UAV position.

Figure 4 shows the desired trajectory for each UAV in black, and the trajectory achieved by each UAV in distinct colors. Square and ‘*’ markers show respectively the desired and achieved positions in specific and equally spaced time instants. When a ‘*’ is inside the square, the UAV is in its desired position.

**Figure 5:**
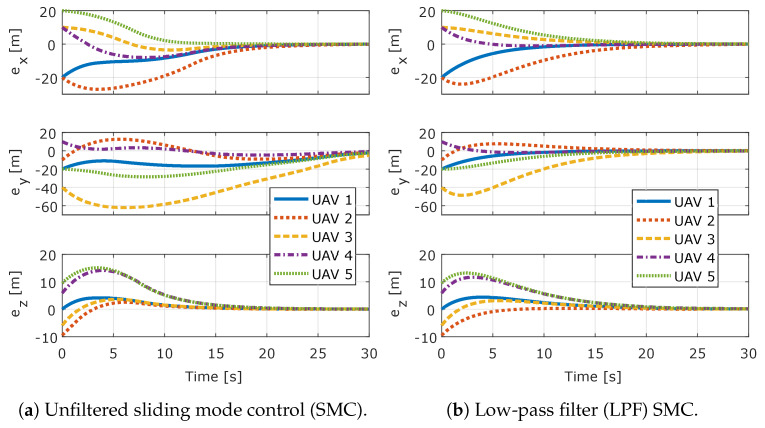
Position errors for all UAVs.

**Figure 6:**
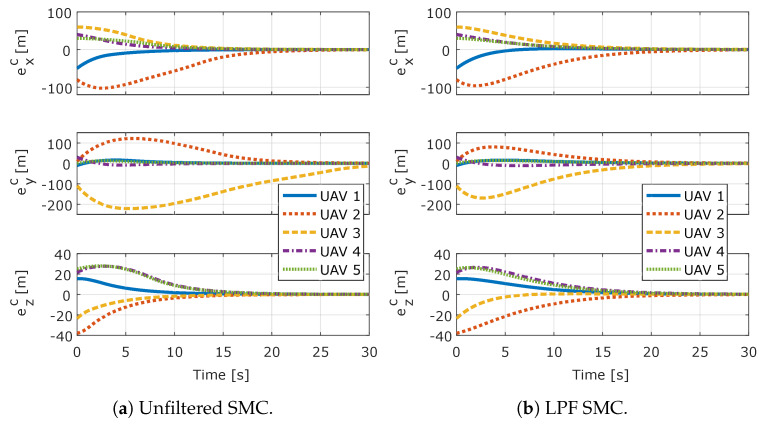
Coupled errors for all UAVs.

Figure 5 shows the formation flight error components $e_{xi}$, $e_{yi}$, and $e_{zi}$ for each *i*-th UAV for both controllers. Figure 6, shows the coupled error of each *i*-th UAV, which is given by Equation (46) for both controllers. It can be seen that, for both controllers, the system rapidly enters in sliding mode, the coupled errors slide in the prescribed linear sliding surface and achieve the performance described by the linear system that defines the sliding surface. It can also be seen that the error converges to zero, which shows that both controllers completely compensate for the added input disturbance.

**Figure 7:**
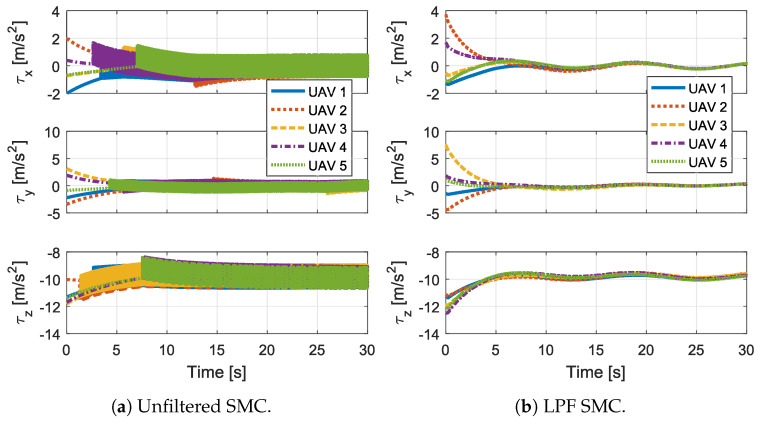
The controller output for all UAVs.

Figure 7 shows the controller output $\tau_{xi}$, $\tau_{yi}$, and $\tau_{zi}$ for each *i*-th UAV, which is generated by adding the smooth $\tau_{i}^{s}$ control signal and $\tau_{i}^{f}$, obtained by filtering the chattering signal $U_{i}$ in the proposed controller, or is the unfiltered control signal in the controller from Reference [18]. As can be seen, the proposed control output is smooth, whereas the control output from the unfiltered SMC chatters.

## 5. Conclusions

A decentralized architecture for synchronous formation flight of UAVs based on sliding mode control with a low pass filter was proposed. The use of the SMC technique provides robustness to disturbances, in a way that the system slides in the prescribed sliding surface even in the presence of disturbances. The LPF virtually removes the chattering while maintaining the convergence to a null error in steady-state. In the proposed architecture only the chattering component of the control signal is filtered. As a result, the controller has a simpler expression when compared to recent results of the literature, such as in [23]. Also, it is presented an equation that is used to compute the upper bounds in the disturbance and in its derivative to a formation described in a global frame. This equation assumes that the upper bounds are known in the wind frame of each follower UAV. It is proved that the proposed controller is stable, achieving a prescribed sliding surface in finite time.

For future work, more realistic models for UAV and wind gusts can be implemented. Also, it is desired to implement other SOSMC, such as presented in References [21,22], in the context of the synchronous formation flight.

## References

[1] Zheng Z., Qian M., Li P., Yi H. (2019). Distributed Adaptive Control for UAV Formation with Input Saturation and Actuator Fault. IEEE Access.

[2] Cai Z., Wang L., Zhao J., Wu K., Wang Y. (2019). Virtual target guidance-based distributed model predictive control for formation control of multiple UAVs. Chin. J. Aeronaut..

[3] Zhao H., Wu S., Wen Y., Liu W., Wu X. (2019). Modeling and Flight Experiments for Swarms of High Dynamic UAVs: A Stochastic Configuration Control System with Multiplicative Noises. Sensors.

[4] Liao F., Teo R., Wang J.L., Dong X., Lin F., Peng K. (2017). Distributed Formation and Reconfiguration Control of VTOL UAVs. IEEE Trans. Control Syst. Technol..

[5] Park C., Cho N., Lee K., Kim Y. (2015). Formation Flight of Multiple UAVs via Onboard Sensor Information Sharing. Sensors.

[6] He L., Bai P., Liang X., Zhang J., Wang W. (2018). Feedback formation control of UAV swarm with multiple implicit leaders. Aerosp. Sci. Technol..

[7] Brandão A.S., Sarcinelli-Filho M. (2016). On the Guidance of Multiple UAV using a Centralized Formation Control Scheme and Delaunay Triangulation. J. Intell. Robot. Syst..

[8] Singh S.N., Zhang R., Chandler P., Banda S. (2003). Decentralized nonlinear robust control of UAVs in close formation. Int. J. Robust Nonlinear Control.

[9] Rezaee H., Abdollahi F., Talebi H.A. (2014). $H_{\infty }$ based motion synchronization in formation flight with delayed communications. IEEE Trans. Ind. Electron..

[10] Lin W. (2014). Distributed UAV formation control using differential game approach. Aerosp. Sci. Technol..

[11] Li Z., Xing X., Yu J. (2015). Decentralized output-feedback formation control of multiple 3-DOF laboratory helicopters. J. Frankl. Inst..

[12] Ren W. (2007). Consensus strategies for cooperative control of vehicle formations. IET Control Theory A.

[13] Oh K.K., Park M.C., Ahn H.S. (2015). A survey of multi-agent formation control. Automatica.

[14] Cordeiro T.F.K., Ferreira H.C., Ishihara J.Y. Non linear controller and path planner algorithm for an autonomous variable shape formation flight. Proceedings of the 2017 International Conference on Unmanned Aircraft Systems (ICUAS).

[15] Liu W., Gao Z. (2020). A distributed flocking control strategy for UAV groups. Comput. Commun..

[16] Qiu H., Duan H. (2020). A multi-objective pigeon-inspired optimization approach to UAV distributed flocking among obstacles. Inf. Sci..

[17] Campa G., Gu Y., Seanor B., Napolitano M.R., Pollini L., Fravolini M.L. (2007). Design and flight-testing of non-linear formation control laws. Control Eng. Pract..

[18] Cordeiro T.F.K., Ferreira H.C., Ishihara J.Y. Robust and Synchronous Nonlinear Controller for Autonomous Formation Flight of Fixed Wing UASs. Proceedings of the 2019 International Conference on Unmanned Aircraft Systems (ICUAS).

[19] Yu J., Dong X., Li Q., Ren Z. (2018). Time-varying formation tracking for high-order multi-agent systems with switching topologies and a leader of bounded unknown input. J. Frankl. Inst..

[20] Slotine J.J., Li W. (1990). Applied Nonlinear Control.

[21] Mei K., Ding S. (2018). Second-order sliding mode controller design subject to an upper-triangular structure. IEEE Trans. Syst. Man Cybern. Syst..

[22] Ding S., Park J.H., Chen C.C. (2020). Second-order sliding mode controller design with output constraint. Automatica.

[23] Zhao D., Li C., Zhu Q. (2011). Low-pass-filter-based position synchronization sliding mode control for multiple robotic manipulator systems. Proc. Inst. Mech. Eng. I J. Syst. Control Eng..

[24] Chong S., Sheng Y., Xiangyuan Z., Liu X. An improved chattering-free sliding mode control with finite time convergence for reentry vehicle. Proceedings of the 2016 IEEE Chinese Guidance, Navigation and Control Conference (CGNCC).

[25] Balamurugan S., Venkatesh P., Varatharajan M. (2017). Fuzzy sliding-mode control with low pass filter to reduce chattering effect: An experimental validation on Quanser SRIP. Sādhanā.

[26] Xu B., Li J., Yang Y., Wu H., Postolache O. (2020). A Novel Sliding Mode Control with Low-Pass Filter for Nonlinear Handling Chain System in Container Ports. Complexity.

[27] Han T., Guan Z.H., Wu Y., Zheng D.F., Zhang X.H., Xiao J.W. (2016). Three-dimensional containment control for multiple unmanned aerial vehicles. J. Frankl. Inst..

[28] Anderson J.D. (1988). Introduction to Flight.

[29] Stevens B.L., Lewis F.L. (2003). Aircraft Control and Simulation.

